# Effects of plant-microbial combined remediation on soil microbial communities in coal mine dump

**DOI:** 10.3389/fmicb.2026.1767455

**Published:** 2026-04-15

**Authors:** Hanting Qu, Pengfei Wang, Xinyan Liu, Jingpeng Li, Jiajia Xu, Shuming Fan, Jie Liu, Jiaqi Liu, Yuxin Guo, Peng Zhang, Haijing Liu, Yuying Bao

**Affiliations:** 1 Key Laboratory of Forage and Endemic Crop Biotechnology, Ministry of Education, School of Life Sciences, Inner Mongolia University, Hohhot, China; 2 State Key Laboratory of Reproductive Regulatory and Breeding of Grassland Livestock, Inner Mongolia University, Hohhot, China

**Keywords:** coal mine dump ecological restoration, ecosystem multifunctionality, high-throughput sequencing, plant-microbial remediation, soil microbial community stability

## Abstract

Open-pit coal mining in grassland ecosystems causes severe soil degradation and disrupts the native soil microbial communities. However, current remediation research predominantly focuses on plant growth or soil physicochemical properties, with a limited systematic analysis of the soil microbial community during remediation in the grassland coal mine dump. To address this gap, this study used high-throughput sequencing integrated with field experiments to systematically evaluate the effects of plant-microbial combined remediation on soil microbial communities in a coal mine dump located in a fragile, severely cold grassland. Our study revealed divergent restoration of soil bacteria and fungi. Bacterial communities demonstrated a strong recovery capacity, with diversity nearly restored to natural levels, while fungal communities remained significantly suppressed. Restoration treatments, especially AMF inoculation, successfully altered overall microbial structure and increased the abundance of key marker taxa. Network analysis further showed that remediation drove bacterial communities toward greater complexity and cooperation, whereas fungal communities responded with increased modularity. Critically, the assembly of the entire soil microbial community was primarily governed by a total phosphorus gradient, which clearly discriminated between bare dump, natural grassland, and restoration plots. Mixed planting fostered the most resilient bacterial community; however, microbial inoculation within this system proved counterproductive. Fungal resilience remained consistently lower than that of bacteria. Crucially, inoculation fundamentally altered ecosystem multifunctionality in monocultures, demonstrating its essential role in functional enhancement. Our results demonstrate that integrating specific plant combinations with microbial inoculation is key to enhancing soil microbial stability and ecosystem multifunctionality during restoration. In summary, our findings reveal the distinct response of soil microbial communities under remediation, and provide critical insights for the ecological restoration of mining areas.

## 1 Introduction

Open-pit coal mining, while economically important, has resulted in profound and multi-faceted ecological degradation, particularly in sensitive grassland ecosystems (Feng et al., 2024; Ma et al., 2023; Zhang C. et al., 2025). The process involves complete removal of vegetation and excavation of topsoil, leading to immediate disruption of soil stratification and loss of soil integrity (Burger et al., 2023). This physical disturbance triggers a cascade of ecological consequences: the destruction of soil aggregates accelerates organic matter mineralization and depletes soil organic carbon stocks, thereby compromising nutrient retention capacity and water-holding potential (Dong et al., 2025; Xue et al., 2025; Zhang et al., 2023). Furthermore, the disruption extends to microbial communities, altering both the diversity and functional composition of bacterial and fungal assemblages that are fundamental to biogeochemical cycling (Jing et al., 2020; Li et al., 2019). In typical steppe regions, where aboveground biodiversity and belowground ecological processes exhibit tight coupling, these perturbations are particularly severe (Bardgett et al., 2005). The removal of perennial grass species disrupts photosynthetic capacity and reduces root exudation, thereby limiting carbon inputs essential for sustaining soil food webs. Soil compaction from heavy machinery decreases porosity and aeration, creating anaerobic microsites that further shift microbial community structure toward stress-tolerant but functionally limited taxa (Jing et al., 2020). The loss of mycorrhizal networks, especially arbuscular mycorrhizal fungi (AMF), impairs nutrient acquisition and water uptake for remaining vegetation, creating feedback loops that hinder natural recovery (Coban et al., 2022). Concurrently, mining operations expose subsurface strata, leading to pyrite oxidation and acid mine drainage—processes that subsequently alter soil physicochemical properties. This acidification exacerbates heavy metal mobilization while inhibiting nutrient availability, placing additional stress on biological communities (Nordstrom, 2011). The cumulative effect is a breakdown in ecosystem multifunctionality with reduced nutrient cycling capacity, impaired water regulation, and loss of habitat complexity (Chen et al., 2023). Restoring these degraded landscapes requires addressing intertwined challenges: reconstructing soil profiles, reestablishing microbial functional groups, and reassembling plant communities with appropriate traits for stress tolerance and ecosystem engineering (Canarini et al., 2021; Coban et al., 2022; Sun et al., 2025). Rehabilitation often requires more than mere revegetation to reestablish critical biogeochemical interfaces (Sun et al., 2025). However, achieving such integrated recovery remains exceptionally challenging due to the potential for persistent state shifts and the loss of ecological memory in severely degraded soils (Canarini et al., 2021).

Conventional ecological restoration of dump sites has primarily relied on physical leveling, chemical amendments such as applying gypsum for pH adjustment, and direct vegetation seeding (Tordoff et al., 2000). Although these measures can preliminarily improve site conditions, they often fall short of rapidly re-establishing a self-sustaining ecosystem, primarily because they fail to address the fundamental loss of microbial diversity and functional complexity (Chen et al., 2020a; Selvarajan et al., 2022). Indeed, microbial communities are essential for soil health and ecosystem functioning, playing key roles in organic matter decomposition, nutrient transformation (Miao et al., 2025; Zhang M. et al., 2025; Zheng et al., 2025), and the establishment of plant-microbe interactions. These interactions, in turn, facilitate plant growth (Liu et al., 2025). However, in open-pit coal mine dumps, the native microbial communities are often severely disrupted due to soil compaction, contamination, and the loss of organic substrates (Chen et al., 2020b). Among these disrupted communities, AMF, which form fundamental symbiotic alliances with most terrestrial plants, are particularly affected (Zhu et al., 2025). These fungi serve as a critical link between plant roots and the soil matrix, dramatically enhancing water and nutrient uptake, while plants in return supply them with carbon (Wang et al., 2025; Zeng et al., 2025). Beyond nutrition, AMF also improve plant resilience to abiotic stresses and contribute to soil aggregate stability (Yu et al., 2025). Yet in degraded mine environments, native AMF communities are often severely diminished, creating a major bottleneck to natural recovery (Liu et al., 2025). In this context, plant-microbe combined remediation has emerged as an advanced, and highly promising approach to enhance soil fertility, promote vegetation recovery, and stabilize microbial ecosystems (Chi et al., 2025). This synergy occurs because plants provide organic carbon and habitat for microbes (Li et al., 2025), while microbes enhance nutrient availability and stress tolerance for plants (Sharma et al., 2025). However, the application of plant-microbe remediation in grassland mine restoration remains constrained by critical oversights (Huang et al., 2023). Prevailing studies often prioritize reporting descriptive microbial shifts over elucidating the mechanistic links between specific plant-microbe partnerships and the functional reassembly of soil communities (Toju et al., 2018). Furthermore, they frequently overlook the extreme abiotic filters—such as compounded nutrient and moisture stresses in cold-arid climates—that dictate microbial establishment and function in these environments (Evans and Wallenstein, 2014). Most importantly, the short-term focus of existing research fails to distinguish transient changes from the development of stable, self-sustaining microbial networks, leaving long-term restoration efficacy uncertain (Hawkes et al., 2017). Our study is designed to bridge these gaps by investigating how defined remediation strategies drive not only composition but also the functional stability and assembly dynamics of microbial communities under the specific stress regime of a grassland coal mine dump.

This study was conducted in a fragile grassland ecosystem characterized by an extreme and severely cold climate, which presents unique challenges for ecological restoration (Liu et al., 2024; Panmei et al., 2025). Understanding how microbial communities respond to remediation efforts over time is crucial for assessing the long-term sustainability of these strategies (Xue et al., 2025). To address this, we systematically evaluated the effects of plant-microbial combined remediation on soil microbial communities, ecosystem stability, and multifunctionality in a grassland coal mine dump. Specifically, we hypothesized that: (1) plant-microbial combined remediation would significantly increase soil microbial diversity in the coal mine dump, converging toward levels observed in undisturbed grassland ecosystems; (2) remediation treatment would alter microbial community structure, enriching beneficial functional taxa while reducing pollution-tolerant taxa; (3) environmental factors would exhibit significant correlations with microbial community composition, though the direction and strength of these relationships would vary across taxonomic groups; and (4) soil microbial community stability and ecosystem multifunctionality would serve as effective indicators for assessing the outcomes of plant-microbial combined remediation.

## 2 Materials and methods

### 2.1 Study area

The research was conducted at the Shengli No. 1 Open-pit Coal Mine and its surrounding grassland (43°57′–44°14′N, 115°30′–116°26′E) in Xilinhot, Inner Mongolia, China. The area is a semi-arid typical steppe, characterized by extreme seasonal cold temperatures (reaching −25°C in winter) and low annual precipitation (294.74 mm, concentrated in July to September) (Hao et al., 2019; Lei et al., 2016). The native vegetation is dominated by *Stipa grandis*, *Leymus chinensis*, and *Cleistogenes caespitosa*.

### 2.2 Experimental design and sampling

In June 2019, we established a plant-microbial combined remediation experiment on a bare internal dump. The experiment included three revegetation treatments:

(1) *Caragana microphylla* monoculture (A1: non-inoculated; A2: inoculated with AMF);

(2) *C. microphylla* and *Astragalus adsurgens* intercropping (B1: non-inoculated; B2: inoculated with AMF);

(3) *A. adsurgens* monoculture (C1: non-inoculated; C2: inoculated with AMF).

Each experimental plot was a rectangle of 100 × 30 m, a size that facilitated large-scale mechanized farming (Supplementary Figure S1). *C. microphylla* seedlings were planted at a 1 × 1 m spacing, and *A. adsurgens* seeds were sown at 20 g/m^2^. The AMF inoculant (*Funneliformis mosseae*) was applied at 50 g per *C. microphylla* seedling and 20 g/m^2^ for *A. adsurgens*. The granular solid inoculant, provided by the Microbial Reclamation Laboratory at China University of Mining and Technology (Beijing), consisted primarily of AM fungal spores, hyphae, and colonized root fragments, with a measured spore density of approximately 26 spores per gram. Two control sites were established: an untreated bare dump (SR) and a natural grassland reference site (NG). The NG site, an undisturbed fenced grassland, was located approximately 2 km west of the mine. The dominant AMF species in its soil are *Glomus reticulatum*, *G. macrocarpum*, and *Septoglomus deserticola*.

In September 2020, a plant survey and soil sampling were performed in each treatment plot following an S-shaped sampling pattern. Five random 1 × 1 m quadrats were established for vegetation and soil sampling. Within each quadrat, we collected soil cores at the 0–20 cm depth following a five-point sampling pattern. The soil samples were processed as follows: one subsample was stored at −80°C for DNA analysis, another was air-dried for physicochemical properties and AMF spore density analysis, and a third was sieved through a 35-mesh sieve for enzyme activity.

### 2.3 Environmental factor measurement methods

Vegetation characteristics, including coverage (PC), species richness (PR), density (PA) and Shannon-Wiener indices (PS), were measured to assess plant community structure. Soil physicochemical properties were analyzed using standard methods: moisture content (SM) by gravimetric drying (105°C to constant weight) (Ma et al., 2021); pH by Palin test SKW500 kit; ammonium nitrogen (AN) by indophenol blue method (625 nm); nitrate nitrogen (NN) by nitrosalicylic acid method; available potassium (AK) by sodium tetraphenylborate method; available phosphorus (AP) and total phosphorus (TP) by molybdenum blue method (660 nm); and total carbon (TC) and total nitrogen (TN) by elemental analyzer (Vario MACRO cube). Soil enzyme activities were measured spectrophotometrically. Alkaline phosphatase (ALP) activity was determined by phenol release (Ma et al., 2021), urease (UE) activity by NH3 -N production (indophenol blue method), and sucrase (SC) activity by 3,5-dinitrosalicylic acid method. All enzyme activities were expressed as μg product g^−1^ soil h^−1^. The AMF spore density was determined by wet-sieving and sucrose centrifugation of soil samples. Stereomicroscopy (Supplementary Figure S1) was used to verify the establishment of the AMF inoculation. Supplementary Table S1 presents the environmental factor analysis results.

### 2.4 High-throughput sequencing analysis

Soil DNA was extracted using the E.Z.N.A.^®^ Soil DNA Kit (Omega Biotek) and quantified via NanoDrop 2000 spectrophotometry. The V3-V4 region of bacterial 16S rRNA genes (primers 338F/806R) (Parada et al., 2016) and fungal ITS region (primers ITS1F/ITS2R) (Adams et al., 2013) were amplified in triplicate 20 μL reactions containing 4 μL 5 × FastPfu buffer, 2 μL dNTPs, 0.8 μL each primer, 0.4 μL FastPfu polymerase, and 10 ng template DNA. Thermal cycling conditions included 95°C for 3 min; 27 cycles of 95°C for 30 s, 55°C for 30 s, 72°C for 45 s; and final extension at 72°C for 10 min. Purified amplicons were pooled in equimolar and paired-end sequenced on an Illumina MiSeq PE300 platform/NovaSeq PE250 platform (Illumina, San Diego, United States) according to the standard protocols by Majorbio Bio-Pharm Technology Co., Ltd. (Shanghai, China). The raw reads were deposited into the NCBI Sequence Read Archive (SRA) database (Accession Number: PRJNA1300456 and PRJNA1300492 for bacterial and fungal communities, respectively).

Raw sequencing reads were quality-filtered (Q20 over 50 bp sliding windows) and assembled ( ≥ 10 bp overlaps, maximum 0.2 mismatch ratio) using fastp (v0.20.0) and FLASH (v1.2.7) (Chen, 2025; Magoč and Salzberg, 2011). Reads < 50 bp or containing ambiguous bases were discarded. Demultiplexing was performed with exact barcode matching and ≤ 2 nucleotide primer mismatches. Operational taxonomic units (OTUs) with a 97% similarity cut-off were clustered using UPARSE version 7.1, and chimeric sequences were identified and removed (Edgar, 2013; Stackebrandt and Goebel, 1994). The taxonomy of each OTU representative sequence was analyzed by RDP Classifier version 2.2 against the 16S rRNA and ITS databases using a confidence threshold of 0.7 (Wang et al., 2007). To minimize noise and the influence of spurious taxa, the OTU table was subjected to a stringent filtering pipeline. First, sequences assigned to mitochondria, chloroplasts, or those with no taxonomic assignment were removed. Subsequently, a prevalence filter was applied to retain only OTUs present in at least 10% of the samples within any treatment group. An abundance filter then removed OTUs with a mean relative abundance across all samples below 0.01%. Finally, singletons and doubletons were eliminated. All downstream ecological and statistical analyses were performed using this high-confidence, filtered OTU table.

### 2.5 Statistical analysis

Statistical analyses were conducted using SPSS (version 19) for one-way ANOVA with Tukey’s *post hoc* test (significance threshold *P* < 0.05). Beta-diversity was assessed using Bray-Curtis dissimilarity and visualized via principal coordinate analysis (PCoA) with the *vegan package* in R 4.1.0 (R Core Team, 2016). The top 100 most abundant microbial taxa (log10-transformed) were phylogenetically reconstructed using maximum likelihood (1,000 bootstraps) in MEGA X, with tree annotation and visualization conducted in iTOL^1^ (Chen et al., 2020b; Letunic and Bork, 2019). Microbial community patterns were further examined using Linear Discriminant Analysis Effect Size (LEfSe) to identify differentially abundant taxa. Co-occurrence networks were constructed using the iNAP platform^2^ based on SparCC correlation analysis (|ρ| > 0.6, *p* < 0.05). To evaluate the stability and structural rationality of networks, we conducted robustness and vulnerability analyses (Yuan et al., 2021). Key topological features were then calculated to characterize network architecture. Final network visualizations were generated and optimized using Gephi (v0.10.1). Variance inflation factor (VIF) analysis and redundancy analysis (RDA) were implemented in R to assess multicollinearity and environmental drivers, respectively (Wei et al., 2018). Ecosystem stability was quantified through resilience (Orwin) by Shannon diversity of microorganisms to evaluate disturbance responses (Orwin and Wardle, 2004). *Resilience*=2|*D*_0_|/(|*D*_0_| + |*D*_*x*_|)−1, where *D*_0_ is the difference between the control (*C*_0_) and the disturbed soil (*P*_0_) at the end of the disturbance (*t*_0_), and *D*_*x*_ is the difference between the control (*C*_*x*_) and the disturbed soil (*P*_*x*_) at the time point (*t*_*x*_) chosen to measure resilience. Microbial diversity at *t*_0_ (2017, post-disturbance) was determined from the same SR and NG sampling sites used in the current study (*t*_*x*_). The Shannon diversity indices at *t*_0_ were as follows. For bacteria, SR was 8.701 ± 0.184 and NG was 8.518 ± 0.044. For fungi, SR was 6.363 ± 0.028 and NG was 7.498 ± 0.141. Ecosystem multifunctionality (EMF) was assessed by integrating 6 key indicators: AM fungal spore density (symbiotic potential), available phosphorus (P supply), nitrate nitrogen (N cycling efficiency), total carbon (C storage), plant cover (vegetation recovery), and soil moisture (hydrological regulation) (Byrnes et al., 2014; Prach, 2011). EMF was calculated by averaging the standardized (z-scored) values of these six key indicators. Each variable was standardized across all samples before averaging to ensure equal weighting. Data visualization was performed using the Majorbio Cloud Platform2^3^ and ImageGP,3^4^ with final graphical refinement conducted in Adobe Illustrator CS6.

## 3 Results

### 3.1 Differential responses of soil bacterial and fungal diversity and resilience stability to remediation

To comprehensively evaluate the effectiveness of plant-microbial combined restoration in a mining dump ecosystem, we first characterized the overall structure of the soil microbial communities. High-throughput sequencing of composite samples from natural grassland (NG), bare dump (SR), and artificially restored plots revealed bacterial and fungal lineages, establishing a foundational dataset for assessing restoration-induced shifts. After quality control, a total of 3,040,963 raw reads were generated from 24 composite samples, comprising 1,433,160 bacterial reads (average 59,715 per sample) and 1,607,803 fungal reads (average 66,992 per sample). To ensure comparative analysis, we normalized the dataset by randomly selecting 35,246 bacterial and 49,554 fungal reads, corresponding to the lowest number of sequences detected across samples. The rarefaction curves plateaued, indicating sufficient sequencing depth to capture microbial diversity (Supplementary Figure S2). High-throughput sequencing identified 36 bacterial phyla with 872 genera and 13 fungal phyla with 333 genera (Supplementary Figure S3). The main bacterial phyla included Actinobacteria (20.12–54.70%), Proteobacteria (15.70–37.36%), Chloroflexi (8.15–12.74%), Acidobacteria (3.38–11.08%), and Bacteroidetes (1.27–11.21%). The main fungal phyla included Ascomycota (46.73–88.41%), Mortierellomycota (1.49–46.91%), Basidiomycota (0.61–25.82%), unidentified phyla (1.10–19.36%), and Glomeromycota (0.01–3.60%).

**FIGURE 1:**
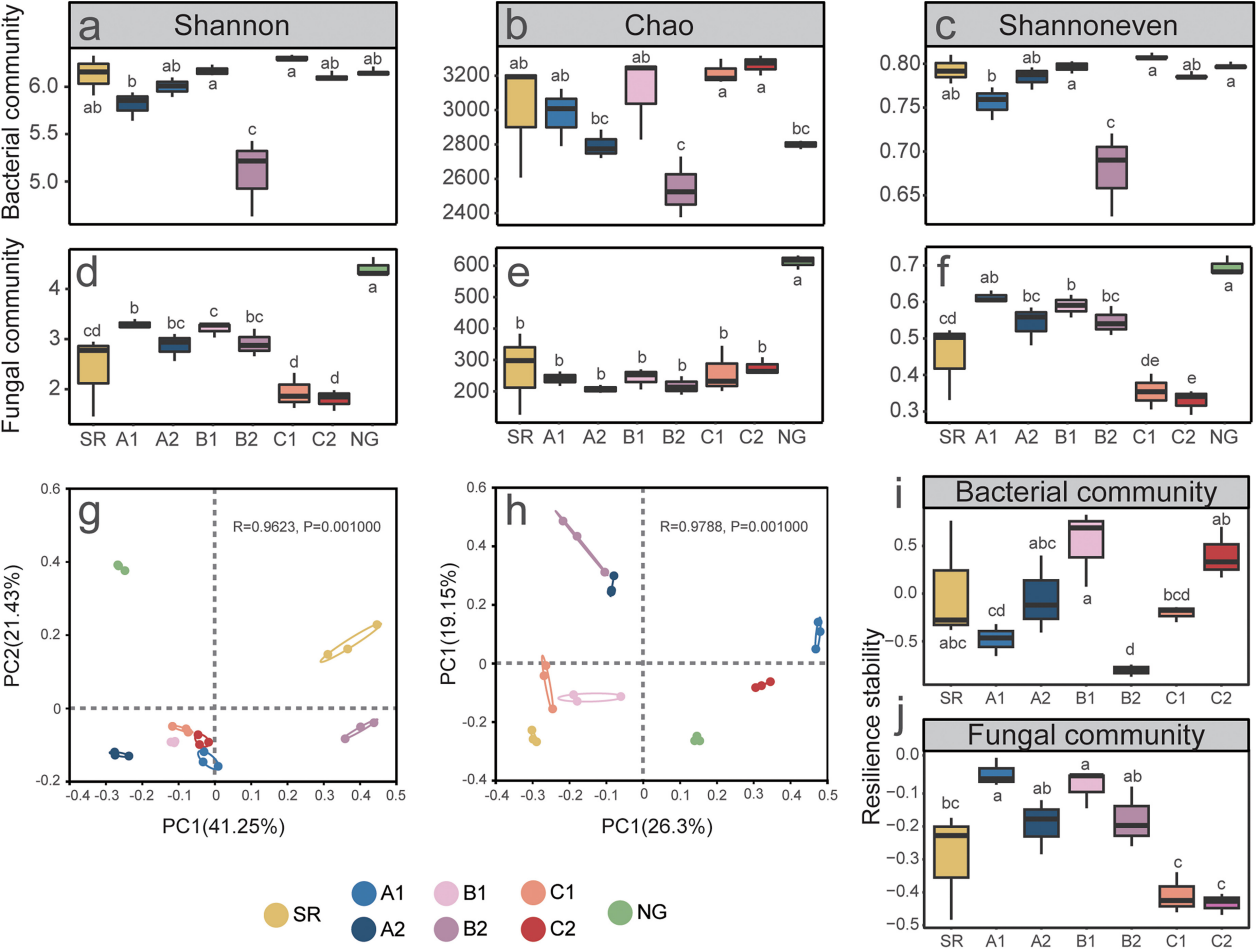
Differential responses of soil bacterial and fungal diversity and resilience stability to remediation. **(a–c)** α-diversity indices of bacterial communities, where **(a)** is Shannon index, **(b)** is Chao index, and **(c)** is Shannoneven index. **(d–f)** α-diversity indices of fungal communities, where **(d)** is Shannon index, **(e)** is Chao index, and **(f)** is Shannoneven index. Principal Component Analysis (PCoA) plot of bacterial communities **(g)** and fungal communities **(h)** based on β-diversity. Resilience stability of bacteria **(i)** and fungi **(j)**. Different letters (a, b, c, etc.) in the figures indicate significant differences between groups (*P* < 0.05).

Microbial restoration patterns diverged between bacteria and fungi (Figure 1). Bacterial communities in the dump (except for B2) approached the diversity levels of the NG (Shannon: SR 6.13 vs. NG 6.14), whereas B2 showed distinct suppression, with 34% lower diversity compared to NG (Figures 1a–c). The Shannon diversity index in B1 was substantially higher than that in B2, with a very large effect size (Cohen’s *d* = 3.67, pooled SD = 0.29). Fungal communities displayed stronger sensitivity to the treatment, with limited recoverability. Both SR and artificial treatments remained below NG (Shannon 4.41). This was consistently evidenced by very large effect sizes across all three metrics between NG and SR: the Shannon index (Cohen’s *d* = 3.42, pooled SD = 0.59), the Chao1 richness estimator (Cohen’s *d* = 3.65, pooled SD = 94.00), and the Shannon evenness (Cohen’s *d* = 3.26, pooled SD = 0.08). The C1 and C2 plots exhibited significantly lower fungal diversity and evenness compared to the NG plot (*P* < 0.05), representing reductions of 56–59% and 50–53%, respectively (Figures 1d–f). Beta diversity analysis revealed distinct microbial community compositions across study areas. The first two PCoA axes explained 62.7 and 45.5% of variance in bacterial and fungal community, respectively. Bacterial communities in A1, B1, C1, and C2 clustered together, while A2 and B2 were separated on opposite sides (Figure 1g). Fungal communities displayed distinct clustering patterns: SR, B1, and C1 grouped in the lower-left quadrant; A2 and B2 in the upper-left; and NG, A1, and C2 scattered on the right side (Figure 1h). NG and SR were distinct from artificially restored plots along PC1. This study demonstrates a fundamental divergence in restoration trajectories between soil bacteria and fungi under plant-microbial remediation. Bacterial communities exhibited a stronger capacity for recovery, nearly reaching natural diversity levels in most sites. In contrast, fungal communities were significantly more sensitive to disturbance and largely failed to rebound, remaining markedly suppressed across all restoration treatments.

Soil microbial community resilience was calculated based on the Shannon diversity index. By comparing the stability patterns of bacterial and fungal communities under different treatments in response to disturbances, we aimed to elucidate the regulatory role of plant-microbial combined remediation on soil microbial community stability. Significant differences in bacterial resilience stability were observed among the different vegetation restoration treatments and SR (Figure 1i). B1 exhibited the highest bacterial resilience stability (0.5293), which was significantly higher than that of SR (0.036) and most other treatments (*p* < 0.05). In contrast, B2 showed the lowest bacterial resilience stability (−0.8037), indicating a negative effect of inoculation under this planting pattern. C2 demonstrated a relatively high stability (0.4007), while A1 showed the lowest value among monocultures (−0.4793). Fungal communities were less resilient than bacterial communities under the studied conditions (Figure 1j). SR showed an intermediate fungal resilience stability (−0.295). Among the restoration treatments, A1 and B1 exhibited the highest fungal resilience (−0.0402 and −0.0787, respectively), which were significantly higher than those of C1, C2 and SR. Both C1 and C2 showed the lowest fungal resilience stability (−0.4082 and −0.4336, respectively), indicating that this species, whether inoculated or not, did not enhance fungal community recovery. Bacterial resilience varied significantly across treatments, with B1 being the highest and B2 the lowest, whereas fungal resilience was generally lower, with A1 and B1 performing best and C1/C2 showing the weakest recovery capacity.

### 3.2 Taxon-specific microbial assembly revealed by phylogenetic analysis

**FIGURE 2:**
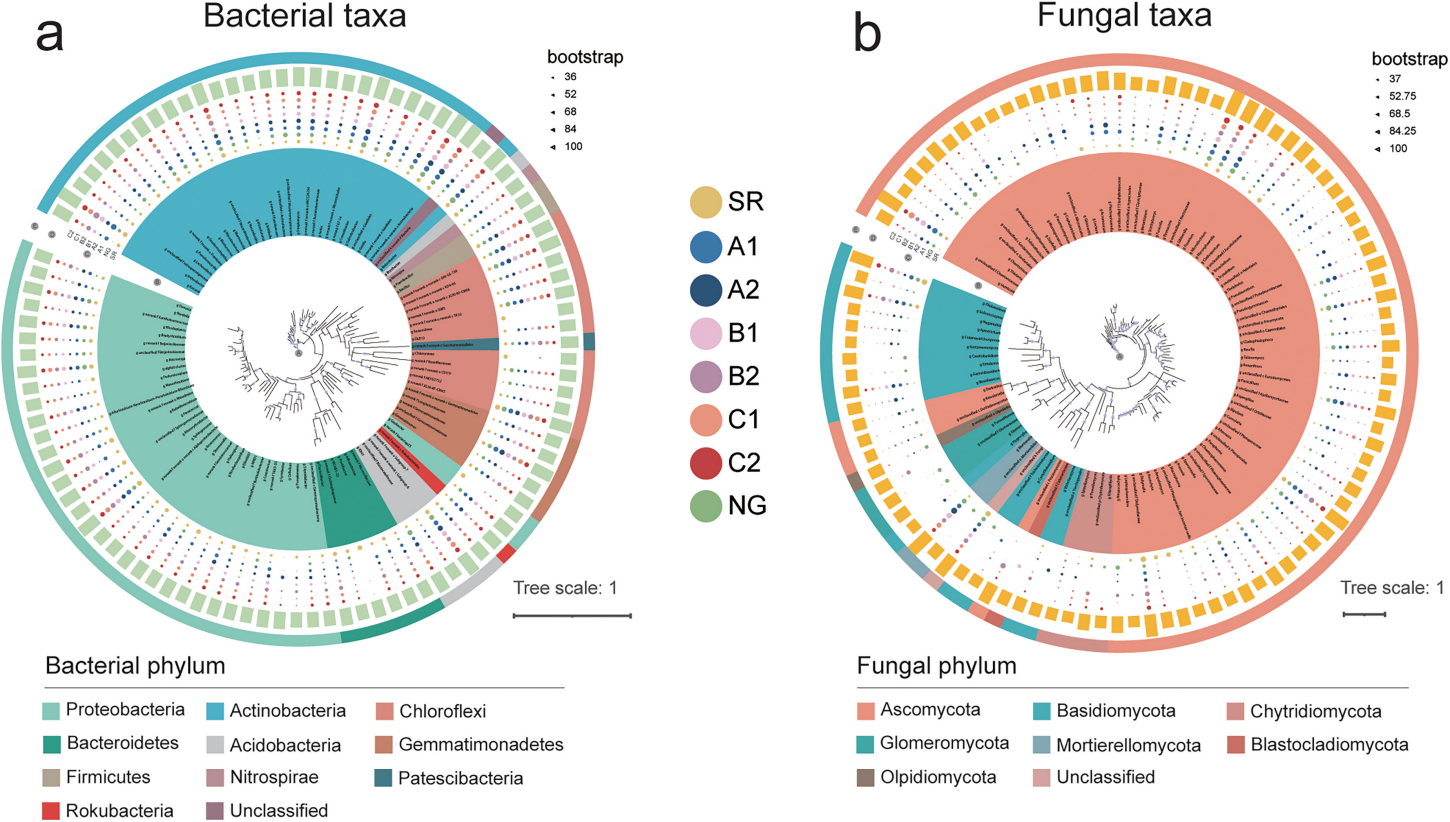
Phylogenetic trees of the top 100 genera showing differences at the study sites (results were visualized using the iTol tool). Relative abundance of the core microbiomes and phylogenetic relationships of bacterial **(a)** and fungal **(b)** are shown. A: Phylogenetic tree constructed using MEGA-X and colored at the phylum level; B: Each genus colored by the phylum level; C: Abundance of each genus is indicated in the outer ring with a shape plot, the size of the circle represents the number of reads per genus; C: Number of reads in each genus for every sample (different colors and circle sizes correspond to the sample and number of reads, respectively); D: Number of reads in each genus for the sum of all samples; E: Each genus and the corresponding phylum. Data were transformed using the natural logarithm (log10).

Building upon this taxonomic inventory, a phylogenetic analysis of the top 100 genera across different plots was conducted to investigate the specific composition and site-specific distribution of soil microbial communities under plant-microbial restoration (Figure 2). In bacterial communities, Proteobacteria was the dominant phylum across all sites, with its relative abundance highest in SR and significantly lower in NG (Figure 2a). Among the revegetation treatments, Proteobacteria abundance showed higher prevalence in Caragana monoculture. Key genera, such as *Sphingomonas* and *Rubellimicrobium*, followed similar trends, while *Geobacter* and *Pseudomonas* were enriched in SR but nearly absent in NG. Actinobacteria exhibited an inverse pattern to Proteobacteria, most abundant in NG and least in SR. Revegetation increased the abundance of Actinobacteria, particularly that of *Arthrobacter* and *Nocardioides*. Chloroflexi displayed intermediate abundance in both NG and SR, and revegetation treatments maintained similar levels to SR. Acidobacteria and Gemmatimonadetes were markedly more abundant in NG than in SR, with revegetation restoring their populations. Bacteroidetes were nearly absent in NG.

In contrast to the relatively uniform distribution of bacteria, fungi exhibited more gaps and variations (Figure 2b). Ascomycota was more abundant in NG than in SR. Among the revegetation treatments, *C. microphylla* monoculture supported a greater abundance of Ascomycota than the others. *Ascochyta* was exclusively found in SR. AM fungal inoculation reduced Ascomycota abundance in most treatments. *Fusarium* exhibited high total abundance. Basidiomycota displayed higher abundance in NG than SR with revegetation increasing its presence. The genera *Filobasidium* and *Naganishia* showed strong recovery with revegetation. Mortierellomycota displayed exceptional patterns, with *Mortierella* exhibiting extremely low abundance in SR but becoming the dominant genus across revegetation treatments. AM inoculation reduced its abundance in B2 and C2. Chytridiomycota exhibited specific distribution, being relatively abundant in both SR and NG but showing variable responses to revegetation. AMF were nearly absent in dump sites except for B2, but were present in NG (relative abundance 2.88–2.93). Notably, the AM fungal composition differed completely between B2 and NG: B2 exclusively contained inoculated *F. mosseae*, whereas NG was dominated by *Glomus*. Revegetation markedly shifted the soil microbial community structure. However, the effectiveness of these restoration treatments varied significantly in reconstituting specific functional groups, such as the composition of AMF.

### 3.3 Microbial biomarkers identified by LEfSe analysis

**FIGURE 3:**
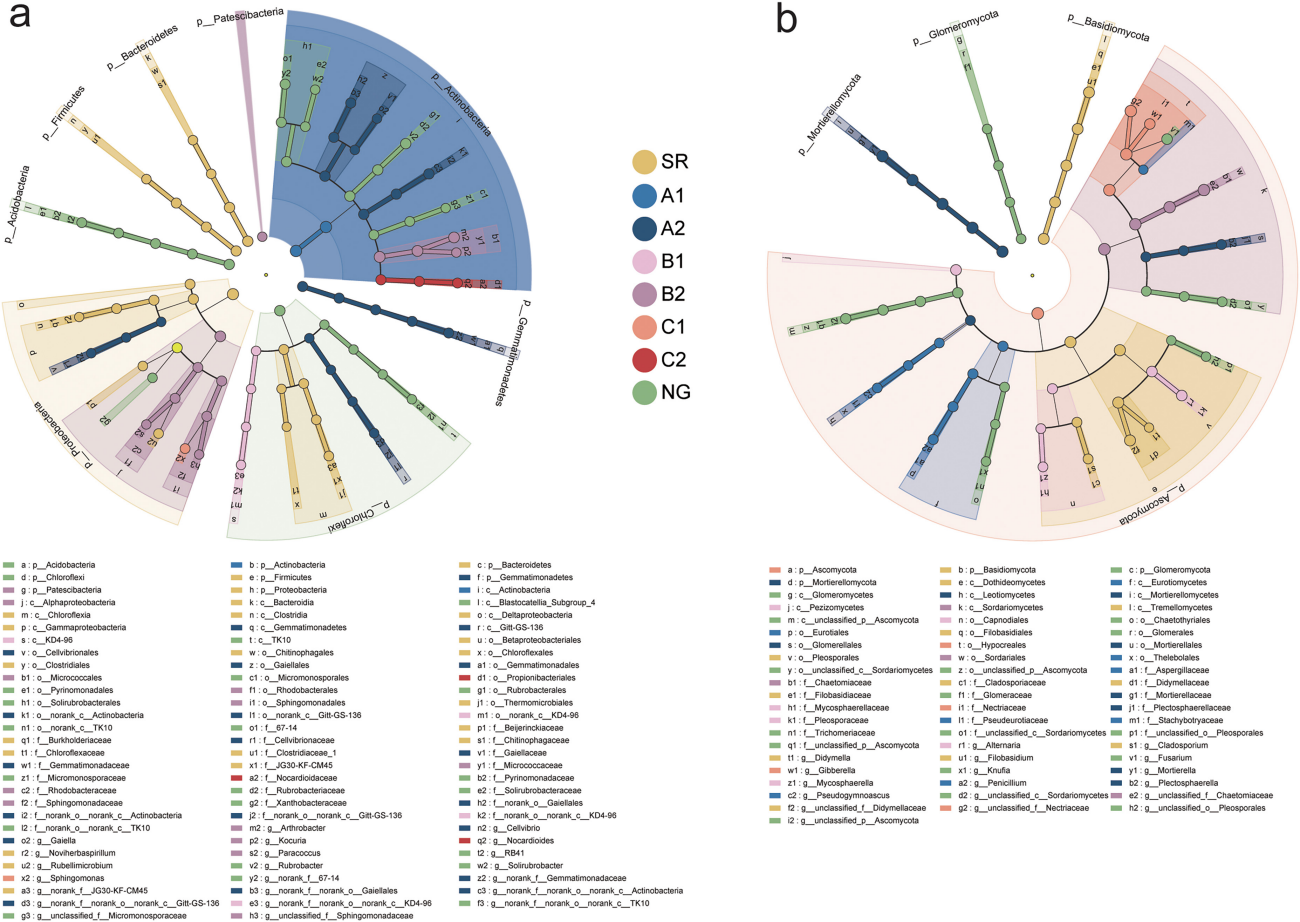
LEfSe of the bacterial **(a)** and fungal **(b)** communities with an LDA score higher than 4.0. Cladograms indicate the phylogenetic distribution of microbial lineages associated with the study sites. Circles represent phylogenetic levels from kingdom to genus.

To further pinpoint the specific microbial taxa that most strongly distinguished each restoration strategy from another, we performed a linear discriminant analysis effect size (LEfSe) analysis. This allowed us to identify robust, statistically significant biomarkers (LDA score ≥ 4) that serve as hallmarks for the unique microbial environments created by each intervention. LEfSe analysis revealed distinct microbial specialization patterns across sites, identifying 86 bacterial clades (8 phyla) and 61 fungal clades (4 phyla) (Figure 3). Notably, fungi demonstrated stronger ecosystem associations, with 18 clades showing significant LDA scores ( > 5) compared to just 5 bacterial clades (Supplementary Figure S4). NG harbored the most complex microbial communities, featuring 22 bacterial and 17 fungal biomarkers, including Rubrobacterales, Solirubrobacterales and Glomeromycota. In contrast, SR developed 21 bacterial and 12 fungal biomarkers, dominated by Chloroflexi, Proteobacteria, and Tremellomycetes. Artificial restoration drove the following trends: (1) intercropping enhanced bacterial biomarker richness but eliminated fungal biomarkers; (2) AMF inoculation consistently increased biomarker numbers, particularly for bacteria (A2: 20; B2: 12) compared to non-inoculated counterparts. Artificial restoration measures effectively altered the structure and marker taxa of the microbial community. AMF inoculation generally increased the abundance of marker species for both bacteria and fungi. These findings reveal the differential regulatory effects of various restoration strategies on the soil microbial community.

### 3.4 Treatment-specific microbial interaction patterns inferred from co-occurrence networks

**FIGURE 4:**
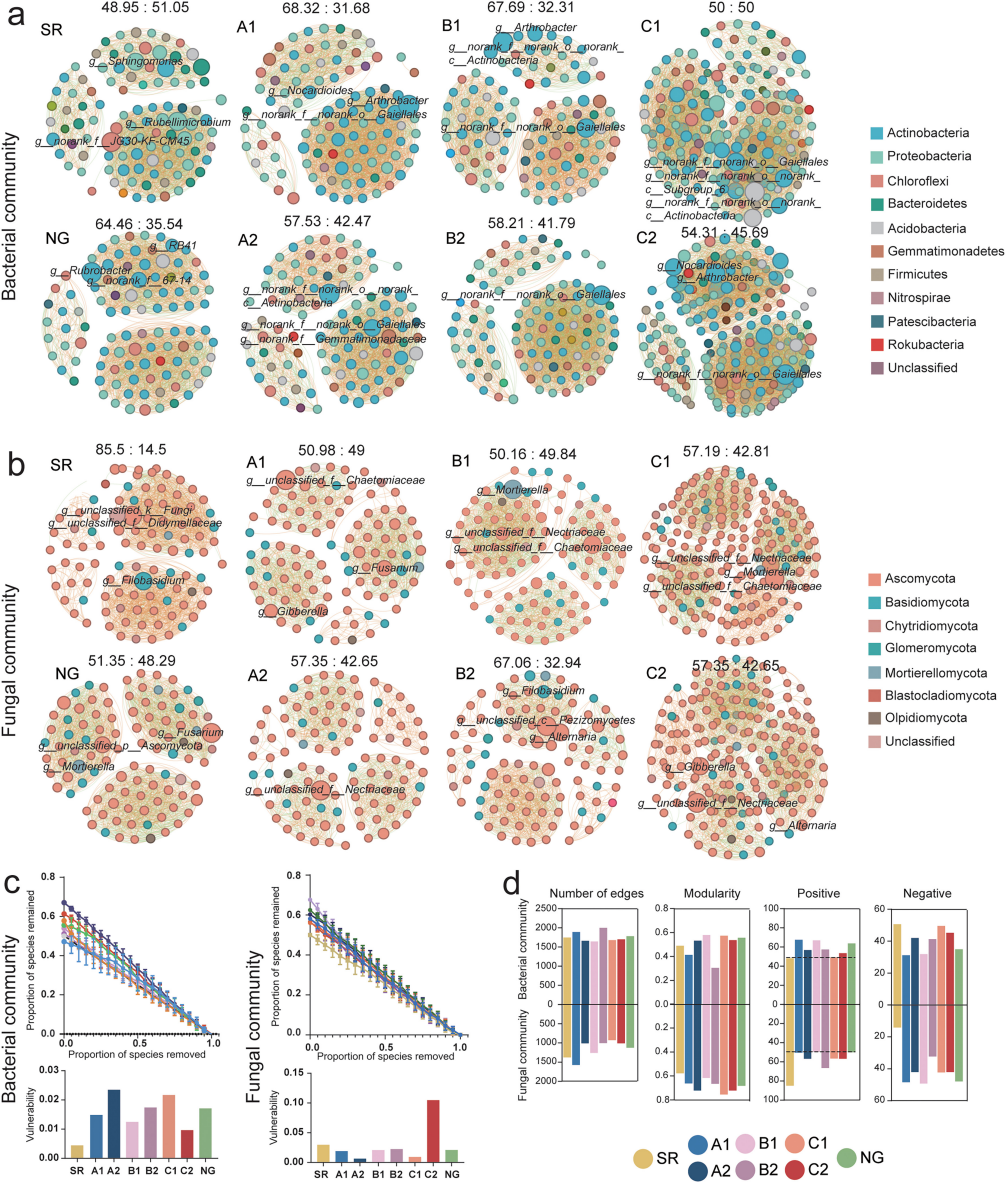
Co-occurrence networks of bacterial **(a)** and fungal **(b)** communities across different treatment groups. In each subfigure, each dot represents an operational taxonomic unit (genus), with color indicating bacterial **(a)** or fungal **(b)** phylum and size corresponding to relative abundance. Notable genera are labeled in each panel. The positive and negative link ratio shown on each network graph. **(c)** The network stability and vulnerability. **(d)** Co-occurrence network properties of bacterial and fungal communities. From left to right: number of edges (network connectivity), modularity (network modular structure), positive (number of positive interactions), and negative (number of negative interactions) across groups.

Beyond mere presence or absence, we sought to understand how these microbial taxa interact within the emerging ecosystem. By constructing a single-factor correlation-based co-occurrence network with the top 100 total abundance at the genus level microorganisms, we decoded the complex web of potential interactions and assessed the topology of the microbial community, providing insights into its stability and complexity under remediation (Figure 4). Bacterial communities were dominated by Actinobacteria and Proteobacteria, with key genera such as *Arthrobacter*, *Rubrobacter*, *Sphingomonas* (Figure 4a). Fungal communities were primarily structured by Didymellaceae, Nectriaceae (Figure 4b). Network stability analyses revealed distinct structural profiles for bacterial and fungal communities (Figure 4c). Bacterial networks in A2 and C2 exhibited the highest robustness (0.6705 and 0.6129, respectively), while A1 was least robust (0.4713). However, vulnerability analysis indicated that the robust bacterial network in A2 was paradoxically fragile (0.0235), whereas C2 maintained both high robustness and low vulnerability (0.0096), suggesting a resilient, decentralized structure. Fungal networks presented an inverse pattern: B1 showed highest robustness (0.6769), but C2 was most vulnerable (0.1046). Bacterial and fungal communities revealed distinct topological patterns across treatments (Figure 4d). For bacterial communities, SR exhibited low connectivity (1,765 edges), while B2 showed the highest network complexity (2017 edges). Positive interactions dominated in NG (64.46%) and most restoration treatments, except SR (48.95%) and C1 (balanced 50:50 ratio). In contrast, fungal networks demonstrated fundamentally different patterns. Restoration generally reduced fungal network complexity compared to SR, while modularity remained elevated across all treatments (0.620–0.725) relative to SR (0.581), peaking in C1 (0.758). SR displayed an unusually high positive edge proportion (85.5%), while NG maintained a balanced ratio (51.35:48.29). AMF inoculation consistently increased the proportion of positive correlations. Soil microbial communities exhibited distinct, treatment-driven assembly network patterns shaped by restoration. Bacterial networks trended toward greater complexity and cooperative interactions, whereas fungal networks increased modularity.

### 3.5 Phosphorus structures microbial communities; inoculation enhances multifunctionality in restoration

**FIGURE 5:**
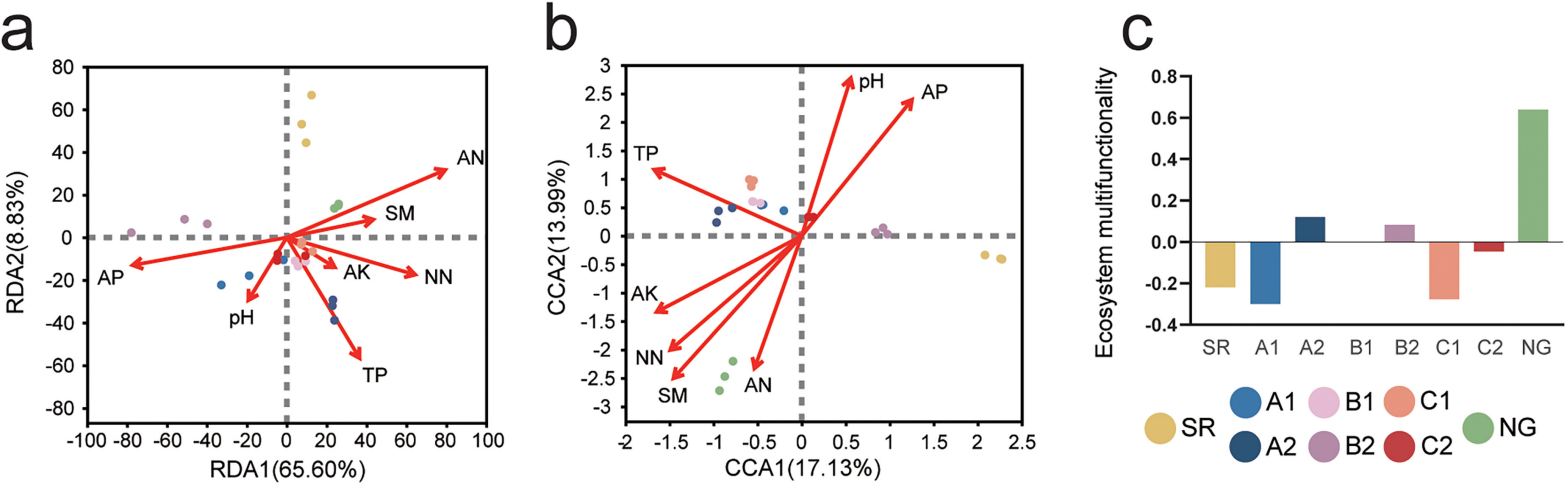
Redundancy analysis (RDA) of the bacterial **(a)** and canonical correspondence analysis (CCA) of the fungal **(b)** community compositions and environmental factors. Circle represents studying sites microbial community. Arrows and words represent different types of environmental factors. SM, moisture content; AN, ammonium nitrogen; NN, nitrate nitrogen; AK, available potassium; AP, available phosphorus; TP, total phosphorus. **(c)** Ecosystem multifunctionality of plant-microbial combined remediation on soil microbial communities.

Recognizing that microbial assembly is driven by both biotic interactions and abiotic constraints, we then interrogated the role of environmental filtering. Key, non-collinear environmental factors were selected using variance inflation factor (VIF) screening, and their constrained influence on the taxonomic structures of both bacterial and fungal communities was quantified through redundancy analysis (RDA). VIF analysis identified seven key environmental drivers (SM, pH, AN, NN, AK, AP, TP). The RDA further revealed distinct microbial community segregation, showing that SR, NG, and B2 formed separate clusters from the other sites (Figure 5). Bacterial communities explained 74.43% of the variance (Figure 5a), while fungal communities accounted for 31.12% (Figure 5b). TP was the dominant environmental factor influencing community assembly, showing negative correlations with SR and B2 but positive associations with other sites. Other sites were all distributed along the positive direction of the TP gradient, while NG exhibited minimal influence. Along the TP gradient, environmental factors bifurcated into opposing groups: pH/AP (associated with B2) versus SM/AN/NN/AK (linked to NG). The assembly of soil microbial communities was primarily driven by a core environmental gradient with TP as the dominant factor. This TP-driven gradient effectively distinguished the SR, NG, and B2, underscoring the pivotal role of phosphorus availability in steering restoration pathways.

To investigate how different vegetation restoration strategies affect ecosystem multifunctionality, we comprehensively assessed EMF by integrating multiple soil and plant functional indicators. This analysis aimed to quantify restoration effectiveness and clarify the role of microbial inoculation in enhancing ecosystem functions. The EMF index varied significantly across the different treatment groups (Figure 5c; *p* < 0.05). NG exhibited the highest EMF value (0.636), which was significantly greater than all restoration treatments and SR, establishing a benchmark for ecosystem functional recovery. In contrast, SR showed low EMF (−0.2189), indicating severely impaired ecosystem functioning. Among the artificial restoration treatments, A2 achieved a high EMF value (0.1197), which was significantly higher than the non-inoculated counterpart (A1, −0.2982). This result highlights the critical role of microbial inoculation in enhancing ecosystem multifunctionality in monoculture plantations of this species. The mixed planting treatments, both B1 and B2, yielded low EMF values (0.0009 and 0.0821, respectively). Although these values were not statistically different from each other, they were significantly higher than those of SR and A1. However, they remained substantially lower than NG and A2. C1 and C2 displayed poor performance in supporting ecosystem multifunctionality. C1 resulted in an EMF value similar to SR (−0.2765). Although C2 led to a significant improvement over C1, it still indicated a failure to restore a positively functioning ecosystem. The results showed that microbial inoculation was essential for enhancing ecosystem multifunctionality in monocultures, with the best-performing inoculated monoculture surpassing mixed planting, while the *A. adsurgens* monoculture was ineffective for restoration under the given conditions.

## 4 Discussion

### 4.1 Limited microbial diversity but altered community structure after restoration

The near-complete recovery of bacterial diversity in most restoration treatments (Figures 1a–c) aligns with previous studies showing rapid bacterial recolonization in disturbed soils (Liang et al., 2020). Bacteria’s shorter generation times and broader dispersal capabilities may explain this resilience (Hawkins and Zeglin, 2022). In contrast, the limited fungal recovery observed here mirrors findings from mining-restoration studies, likely reflecting fungi’s stronger dependence on plant-derived carbon and sensitivity to abiotic stressors (Yu et al., 2025). Although aboveground plant communities differed markedly, our study found that planting or inoculating did not significantly improve microbial α-diversity compared to the bare dump. This surprising result challenges the conventional paradigm that plant establishment should directly promote microbial diversity in degraded soils (Albright et al., 2022). Several explanations may account for this phenomenon. First, the tailings substrate itself may harbor a depauperate microbial species pool (potentially including pathogenic microbial communities) constrained by harsh edaphic conditions (e.g., heavy metals, poor nutrients) (Liang et al., 2020), limiting the potential for plant-mediated microbial recruitment (Albright et al., 2022). Second, the use of monocultures or low diversity planting created homogeneous selection pressures through simplified root exudate profiles and reduced heterogeneity (Yu et al., 2025), as particularly evident in the *A. adsurgens* monocultures which showed reduced fungal diversity. The significant bacterial diversity reduction in B2 plots contrasts with studies reporting neutral or positive AMF-bacteria interactions. This suppression could stem from competitive exclusion via altered resource competition (e.g., AMF monopolizing root exudates) or direct antagonism. β-diversity results corroborate and complement α-diversity findings. The clustering of bacterial communities across treatments (except B2) (Figure 1g) suggests strong environmental filtering (Lv et al., 2025). Unlike the α-diversity results which showed no difference between SR and artificially restored sites, the clear separation along PC2 between SR and NG versus artificially restored sites reveals fundamental differences in microbial community assembly processes (Albright et al., 2022), thereby confirming the substantial impact of artificial restoration on microbial diversity. These findings require further validation and interpretation through subsequent microbial community composition analysis.

### 4.2 Differential microbial responses to vegetation and inoculation in mine restoration

The present study revealed pronounced shifts in soil microbial community composition and structure across different restoration strategies, highlighting the roles of vegetation type and AM fungal inoculation in driving these changes (Harris, 2009). Our findings demonstrate that bacterial and fungal communities respond distinctly to ecological restoration, with implications for ecosystem recovery and stability (Van Der Heijden et al., 2008). Phylogenetic analysis indicated that Proteobacteria dominated bacterial communities in SR (Figure 2a), consistent with their known role of thriving in nutrient-poor and disturbed environments (Fierer et al., 2007). Their significantly lower abundance in NG suggests a shift toward more stable and mature microbial assemblages (Jangid et al., 2008). The enrichment of genera such as *Geobacter* and *Pseudomonas* in SR, both known for their metabolic versatility and stress tolerance, further supports the idea that disturbed sites favor taxa with rapid growth and adaptive capacity (DeAngelis et al., 2011). Conversely, the increased abundance of Actinobacteria in NG and restored sites aligns with their association with more stable, organic-rich soils (Bardgett and Van Der Putten, 2014). The recovery of Acidobacteria and Gemmatimonadetes in revegetated treatments indicates a gradual return to a more complex and functionally diverse soil microbiome (Navarrete et al., 2013). Fungal communities exhibited even greater sensitivity to restoration practices (Figure 2b). The higher abundance of Ascomycota in NG and its selective enrichment in *C. microphylla* monoculture suggest strong plant-fungal interactions, possibly related to litter quality and root exudates (Averill, 2016). Although the spore density provided limited evidence for inoculation effectiveness, the near absence of AMF in dump sites and the distinct compositional difference between NG and B2 underscore the challenge of AMF re-establishment (Koziol and Bever, 2017). The dominance of *Mortierella* in revegetated treatments, a genus known for its role in nutrient cycling and plant growth promotion, highlights the potential for functional recovery through targeted restoration (Ozimek and Hanaka, 2020).

LEfSe analysis further confirmed the strong ecosystem specificity of microbial clades, particularly fungi, which showed higher LDA scores and greater biomarker richness in NG (Figure 3 and Supplementary Figure S4). This suggests that fungal communities may serve as more sensitive indicators of ecosystem recovery than bacteria (Tedersoo et al., 2014). The fact that AMF inoculation consistently increased biomarker richness, especially in bacterial communities, indicates that microbial inoculation can accelerate the reassembly of soil microbiomes, possibly through enhanced plant health and altered soil conditions (Wubs et al., 2016). Notably, Acidobacteria (enriched in NG) and *Mortierella* (enriched in A2) were identified as key markers. These taxa play pivotal, mechanistically distinct roles in soil nutrient cycling, which helps explain the observed soil chemical properties and plant performance. The enrichment of Acidobacteria in natural grassland soils aligns with its well-established ecological strategy as an oligotroph (Kielak et al., 2016). This phylum is adapted to nutrient-poor conditions, exhibiting slow growth rates and high-affinity substrate uptake systems. Their relative dominance under reduced fertilization highlights a microbial community shift toward conservative nutrient use. Importantly, certain subgroups within Acidobacteria are directly implicated in phosphorus mobilization. They possess genes encoding enzymes like acid phosphatases and C-P lyases, enabling them to mineralize organic phosphorus and potentially solubilize inorganic phosphates under acidic conditions, thereby contributing to the plant-available P pool in low-input systems (Dai et al., 2020). Conversely, under specific treatments, the prominence of *Mortierella*, a ubiquitous saprotrophic fungus, can be mechanistically linked to its versatile enzymatic arsenal. *Mortierella* spp. are prolific producers of extracellular phosphatases and phytases, driving the mineralization of organic phosphorus compounds prevalent in manure and compost (Zhang et al., 2016). Furthermore, many *Mortierella* strains are effective phosphate-solubilizers, capable of releasing bound inorganic phosphorus through the secretion of low-molecular-weight organic acids such as gluconic and citric acid. This functional capacity provides a direct microbial mechanism for the enhanced phosphorus availability and uptake often observed with organic fertilization. Its role extends beyond P cycling, as *Mortierella* also participates in carbon turnover and can engage in beneficial interactions with plant roots, potentially enhancing plant growth (Ozimek and Hanaka, 2020). Therefore, the marker taxa identified are not merely statistical signatures but represent functional keystones that underpin the differential nutrient cycling pathways across fertilization regimes.

Network stability metrics transform our understanding from static connectivity to dynamic ecological resilience (Dunne et al., 2002). The high robustness of networks in A2 and C2 indicates architectural buffering against random species loss. Vulnerability analysis provides a crucial complement: the high vulnerability of A2 reveals a paradox—it is resilient to random failure yet fragile to targeted attack on its keystone hubs (Yuan et al., 2021). In contrast, the low vulnerability of C2 reflects a safer, distributed architecture where functional redundancy buffers against perturbation (Loreau and De Mazancourt, 2013). Critically, AMF inoculation elicited divergent stability strategies. In A2, inoculation acted as a structural engineer, concurrently increasing robustness and decreasing vulnerability to build a coherent, perturbation-resistant network. In the C2, inoculation functioned as a risk distributor, not enhancing robustness but significantly reducing vulnerability, steering the network toward a redundancy-based stability strategy. This demonstrates that inoculation’s ecological role is context-dependent, pivoting from network rebuilder in degraded systems to resilience modulator in diverse communities (Rillig et al., 2021). Therefore, the assessment of microbial network stability should inform restoration planning. Network analysis provided additional insights into the topological organization of microbial communities (Figure 4). The higher connectivity and complexity in B2 (AMF inoculated intercropping) suggest that synergistic interactions between plants and microbes enhance microbial co-occurrence and stability (Wang et al., 2024). The dominance of positive correlations in most restored treatments, particularly after AMF inoculation, implies a more cooperative and less competitive microbial network, which may contribute to ecosystem resilience (Hernandez et al., 2021). In contrast, the highly positive but less modular fungal network in SR may reflect a stressed and simplified community structure.

### 4.3 Nutrients drive microbial recovery

The RDA analysis revealed differences in how bacterial and fungal communities respond to environmental factors (Figure 5). Bacterial communities showed remarkably strong structuring by nitrogen and phosphorus availability (AN, NN, AP, TP). This strong nutrient dependence reflects many dominant bacterial taxa and their crucial roles in nutrient cycling processes (Fierer et al., 2012). In contrast, fungal communities exhibited more complex regulation, which aligns with their diverse ecological strategies ranging from oligotrophic decomposers to symbiotic specialists. TP emerged as the primary environmental filter, but with treatment-specific effects that reveal important restoration insights (Delgado-Baquerizo et al., 2016). The strong negative correlation with SR communities confirms severe phosphorus limitation in unrestored tailings, while the positive association in most artificial restoration sites (especially A2) demonstrates successful phosphorus mobilization through revegetation (Richardson and Simpson, 2011). The exceptional position of NG, showing minimal TP responsiveness, likely reflects the balanced, efficient nutrient cycling characteristic of mature ecosystems (Wardle et al., 2004). Notably, the AMF inoculated B2 treatment clustered separately from other restored sites in phosphorus relations, indicating that mycorrhizal inoculation fundamentally alters phosphorus dynamics and consequent microbial assembly patterns (Svenningsen et al., 2018). SM emerged as a critical determinant of microbial community segregation, particularly distinguishing the NG from other sites. The strong positive association of NG with SM reflects the characteristic of mature ecosystems to maintain stable microhydrological conditions through developed root systems and organic matter accumulation (Bach et al., 2010). This moisture stability supports fungal-dominated communities, particularly mycorrhizal networks, which are highly sensitive to water potential fluctuations (Hawkes et al., 2005). Consequently, water stress remains a persistent constraint that significantly limits microbial recovery during the initial stages of ecosystem restoration. The coupling of SM with AN and NN in the RDA ordination suggests that moisture availability mediates nitrogen transformation and uptake, creating an integrated limitation that restoration must address. Soil pH exhibited a distinct regulatory role, forming a key axis with AP that characterized the B2 treatment. This pH-AP coupling in the mycorrhizal-inoculated plots suggests that AMF inoculation may modify rhizosphere pH through organic acid exudation, consequently influencing phosphorus solubility (Li et al., 2024). The minimal pH association with NG communities further supports that in stable ecosystems, biological processes buffer against pH fluctuations (Rousk et al., 2010). AN and NN showed contrasting ecological roles despite both being nitrogen sources. Their strong collinearity with SM positions them within the NG, suggesting that nitrogen availability becomes optimal only when hydrological conditions stabilize. The clustering of AN and NN indicates coordinated nitrogen cycling in the NG, where complete nitrification processes are established (Levy-Booth et al., 2014).

### 4.4 Ecosystem stability and multifunctionality in restoration

The stability of soil microbial communities is a critical indicator of ecosystem resilience, particularly in degraded environments such as bare excavated spoil areas (Delgado-Baquerizo et al., 2016). In this study, we evaluated the resilience stability of bacterial and fungal communities under different vegetation restoration strategies, with or without microbial inoculation, to elucidate the role of plant-microbial combined remediation in modulating microbial community recovery after disturbance. Our findings reveal marked differences in bacterial resilience stability across treatments (Figure 1i). The highest bacterial resilience was observed in mixed planting without inoculation, which significantly surpassed not only the bare dump but also most other restoration regimes. This suggests that a specific plant combination can foster a bacterial community with a strong capacity to recover from perturbation (Wubs et al., 2016). In contrast, the introduction of microbial inoculants under the same mixed planting scheme (B2) substantially reduced bacterial resilience, indicating that inoculation may disrupt native microbial interactions or impose additional stress under certain plant configurations (Wei et al., 2019). This underscores the context-dependency of microbial inoculation outcomes, where plant composition mediates inoculant success (Toju et al., 2018). Fungal communities generally exhibited lower resilience compared to bacterial communities across all treatments, aligning with observations that fungal networks are often slower to recover after disturbance (Crowther et al., 2019). The intermediate fungal resilience in bare dump (SR) implies that spontaneous fungal assembly in degraded dumps does not necessarily yield a highly resilient community (Figure 1j). Notably, the highest fungal resilience was recorded in *C. microphylla* monoculture without inoculation (A1) and the mixed planting without inoculation, both of which outperformed not only SR but also *A. adsurgens* treatments. This indicates that *C. microphylla*, either alone or in combination with *A. adsurgens*, promotes fungal community recovery, whereas *A. adsurgens* appears less conducive to fungal resilience. The consistently low fungal resilience in *A. adsurgens* treatments suggests that this plant species may select for fungal assemblages with limited adaptive capacity or may alter soil conditions in ways that impede fungal recovery (Bever et al., 2010). Specifically, in our cold-climate study site, the slower decomposition rates and limited growing season may amplify the disadvantage of *A. adsurgens* in fostering a resilient fungal network, as AMF are known to exhibit reduced activity and host specificity under low-temperature stress (Jian et al., 2024). The divergent responses of bacterial and fungal communities to the same treatment—exemplified by the contrasting outcomes in B1 (high bacterial but moderate fungal resilience)—reflect the distinct ecological strategies and sensitivities of these microbial groups. Bacteria, with shorter generation times and broader metabolic versatility, may respond more rapidly to plant-derived carbon inputs and soil physicochemical changes, whereas fungi, often dependent on mycorrhizal symbioses and hyphal networks, may be more vulnerable to disruption in plant composition or soil structure (Fierer, 2017). It is important to acknowledge potential methodological biases in our assessment. The use of universal fungal primers may underrepresent key functional groups like AMF, whose ribosomal genes are difficult to amplify with standard primers (Öpik et al., 2010). This could lead to an incomplete picture of the total fungal community and its resilience. Future work incorporating AMF-specific primers or lipid biomarkers would provide a more comprehensive view. In conclusion, our results demonstrate that vegetation restoration strategies significantly alter the resilience stability of soil microbial communities, with effects mediated by plant species composition and microbial inoculation. Mixed planting without inoculation emerged as a promising approach for enhancing both bacterial and fungal resilience, whereas inoculation in mixed systems may compromise bacterial recovery. *A. adsurgens* monoculture, whether inoculated or not, failed to support fungal community resilience. These findings, however, are derived from a short-term study. The initial resilience patterns we observed may shift over time as plant communities mature and soil properties evolve. Long-term monitoring is essential to determine whether the positive effects of mixed planting without inoculation are sustained and whether the initially disruptive effect of inoculation in mixed systems (B2) is transient or persistent (Holl and Aide, 2011). These insights emphasize the importance of tailored plant-microbe combinations in restoration ecology and caution against generalized inoculation practices. Future studies should explore the mechanistic links between plant traits, soil properties, and microbial network dynamics to optimize restoration designs for long-term ecosystem stability (Harris, 2009).

Beyond microbial community stability, ecosystem multifunctionality (EMF) serves as a more integrative measure of restoration success, reflecting the simultaneous performance of multiple soil and plant functions (Bradford et al., 2014). Our assessment of EMF revealed clear hierarchical patterns among treatments, further elucidating the complex outcomes of different restoration strategies (Figure 5c). The significantly higher EMF in the natural grassland benchmark confirms that the target ecosystem state supports a broad suite of tightly coupled functions, against which restoration efforts can be gauged (Allan et al., 2015). In stark contrast, the bare spoil (SR) exhibited severely depressed multifunctionality, quantifying the profound degradation resulting from mining disturbance. The significant enhancement of EMF through AM fungal inoculation provides strong support for the critical role of microbial symbionts in ecosystem recovery (Van Der Heijden et al., 2008). The efficacy of AMF inoculation in our cold-region site, albeit positive in monocultures, may be partially constrained by the environmental conditions. AMF colonization and nutrient transfer efficiency can be suboptimal under low soil temperatures (Latef et al., 2016), suggesting that the observed benefits might be even greater in warmer climates or with cold-adapted AMF strains. This improvement likely stems from multiple mechanisms: (1) enhanced nutrient cycling through fungal hyphal networks (Averill et al., 2014), (2) improved plant health leading to greater organic matter inputs, and (3) stimulation of beneficial soil microbial communities (Wagg et al., 2014). A key finding is the pronounced positive effect of microbial inoculation in monoculture systems. The dramatic increase in EMF from A1 to A2 demonstrates that *C. microphylla* monoculture, when combined with specific microbial consortia, can transition from a functionally net-negative state to one supporting modest but positive multifunctionality. This suggests that the planted host, in isolation, may initially create a constrained rhizosphere environment; inoculation likely alleviates these limitations by introducing key functional traits (e.g., nutrient mobilization, stress tolerance) that unlock the plant’s potential to drive multiple ecosystem processes. However, the limited EMF response in mixed plantings was unexpected. While these treatments improved over SR, their low values indicate that the simple combination of *C. microphylla* and *A. adsurgens* did not create strong complementary or facilitative interactions for boosting multifunctionality under these conditions. This may be due to unresolved interspecific competition or a mismatch between the introduced/preexisting microbes and the two-plant host system (Barry et al., 2019). This striking contrast can be explained by a microbiome-dependent receptivity principle. In monocultures, the microbial niche is functionally constrained, creating a simplified system highly receptive to directional intervention. This explains the decisive shift from net-negative (−0.2982) to positive (0.1197) EMF upon inoculation (A1 vs. A2, Figure 5c). Conversely, diverse mixtures foster a complex and functionally redundant microbiome, which buffers the system against perturbation by a single introduced agent, resulting in the attenuated response observed (Loreau and De Mazancourt, 2013; Wagg et al., 2019). The consistently poor performance of EMF in *A. adsurgens* monoculture indicated that this species, under the given edaphic and climatic constraints, may be an unsuitable candidate for initiating multifunctional recovery (Funk et al., 2017). Its inability to support multifunctionality aligns with its previously noted failure to enhance fungal community resilience, suggesting a species-specific limitation in fostering key belowground processes. In synthesis, these EMF results underscore that: (1) microbial inoculation can be a decisive factor for achieving baseline multifunctionality in monoculture plantations, (2) the choice of plant species is paramount, as some species (e.g., *A. adsurgens*) may be ineffective restoration anchors regardless of mixed planting or microbial management. For scalable restoration protocols, our study suggests a specific ecological restoration strategy: implementing designed plant mixtures, such as intercropping *C. microphylla* with *A. adsurgens* combined with AMF inoculation, which can foster complementary microbial networks in a more cost-effective and low-risk manner than blanket inoculation (Wagg et al., 2019). This advocates for a precision restoration approach, where plant selection and microbial amendments are co-optimized based on functional compatibility, rather than relying solely on plant diversity metrics or generic inoculation practices (Wubs et al., 2016).

## 5 Conclusion

This study systematically evaluated the effects of plant-microbial combined remediation on soil microbial communities in a coal mine dump through integrated field experiments and high-throughput sequencing. Plant-microbe remediation indeed significantly enhanced soil microbial diversity, yet this recovery was taxon-specific. Bacterial diversity demonstrated a remarkable capacity for recovery, nearly converging with levels in undisturbed grasslands. In contrast, fungal diversity remained significantly suppressed, failing to rebound across all treatments. Remediation treatments altered the microbial community structure by enriching beneficial nutrient cyclers and symbiotic bacterial taxa. While bacterial networks trended toward greater complexity and cooperation, fungal communities responded with increased modularity. We found that the assembly of the entire soil microbial community was primarily driven by total phosphorus. This TP-driven gradient effectively distinguished the bare dump, natural grassland, and restoration areas, demonstrating that phosphorus is a master variable that overrides or modulates other relationships. Mixed planting yielded the most resilient bacterial community, while microbial inoculation in this system was counterproductive. Inoculation fundamentally altered the ecosystem multifunctionality of monoculture restoration. Our findings demonstrate that effective restoration of ecosystem multifunctionality requires specific plant-microbe combinations rather than simple plant diversification. Microbial inoculation proved essential for enhancing multifunctionality. We therefore recommend that scalable restoration protocols integrate plant and microbial management, such as through the intercropping of *C. microphylla* with *A. adsurgens* combined with inoculation, to directly enhance microbial stability and ecosystem multifunctionality. These results highlight the importance of targeted microbial management alongside careful plant species selection for successful ecological restoration. This research establishes a critical theoretical foundation for soil reclamation and environmental management in open-pit coal mines from the perspective of soil microbial communities.

## Data Availability

The datasets presented in this study can be found in online repositories. The names of the repository/repositories and accession number(s) can be found at: https://www.ncbi.nlm.nih.gov/, PRJNA1300456; https://www.ncbi.nlm.nih.gov/, PRJNA1300492.

## Footnotes

1
http://itol.embl.de

2
https://inap.denglab.org.cn/

3
https://www.majorbio.com

4
http://www.ehbio.com/ImageGP/
