# Supplementary material for: Effects of plant-microbial combined remediation on soil microbial communities in coal mine dump
Source: Front Microbiol. 2026 Apr 15;17:1767455. doi: 10.3389/fmicb.2026.1767455 (PMC13125021; doi:10.3389/fmicb.2026.1767455)
Supplement: Supplementary file 1 [file Data_Sheet_1.docx]

**Supplementary Materials**

**Effects of plant-microbial combined remediation on soil microbial communities and ecosystem stability in coal mine dump**

Hanting Qu ^a, b^, Pengfei Wang ^a, b^, Xinyan Liu ^a, b^, Jingpeng Li ^a, b^, Jiajia Xu ^a, b^, Shuming Fan ^a, b^, Jie Liu ^a, b^, Jiaqi Liu ^a, b^, Yuxin Guo ^a, b^, Peng Zhang ^a, b^, Haijing Liu ^a, b^, Yuying Bao ^a, b, *^

^a^ Key Laboratory of Herbage and Endemic Crop Biology, Ministry of Education, School of Life Sciences, Inner Mongolia University, Hohhot, 010010, PR China

^b^ State Key Laboratory of Reproductive Regulatory and Breeding of Grassland Livestock, Inner Mongolia University, Hohhot, 010010, PR China

***Corresponding authors:**

Yuying Bao: ndbyy@imu.edu.cn

Tel. Number: 18686099308; Country code: 86 (CHN)

Key Laboratory of Herbage and Endemic Crop Biology, Ministry of Education, School of Life Sciences, Inner Mongolia University, Hohhot, 010010, PR China

Supporting Information Includes:

- 1 Table
- 4 Figures

**Table S1** The results of environmental factor analysis surveyed in the studying areas

| Groups | SM | pH | AN | NN | AK | AP | TP | TC |
| --- | --- | --- | --- | --- | --- | --- | --- | --- |
| SR | 5.00 ± 0.67de | 8.91 ± 0.22bc | 44.92 ± 6.00c | 4.67 ± 0.82e | 10.63 ± 0.50e | 3.23 ± 0.10b | 24.54 ± 3.72de | 3.00 ± 0.04a |
| A1 | 10.64 ± 0.77c | 8.76 ± 0.37c | 9.88 ± 1.35e | 2.81 ± 0.14f | 17.26 ± 1.93c | 3.35 ± 0.11a | 29.97 ± 4.17bcd | 1.69 ± 0.04e |
| A2 | 7.53 ± 1.31d | 9.00 ± 0.13b | 47.97 ± 4.73b | 31.91 ± 0.93b | 18.71 ± 1.14b | 3.06 ± 0.08c | 28.23 ± 2.40cd | 2.22 ± 0.06b |
| B1 | 7.23 ± 0.43d | 8.94 ± 0.24bc | 36.06 ± 4.56d | 3.93 ± 0.46e | 13.59 ± 1.00d | 3.25 ± 0.14b | 37.79 ± 3.59a | 2.14 ± 0.02c |
| B2 | 5.73 ± 0.74e | 8.88 ± 0.12c | 24.01 ± 2.27e | 4.55 ± 0.63e | 16.81 ± 0.86c | 3.44 ± 0.18a | 23.67 ± 2.07e | 1.92 ± 0.02d |
| C1 | 8.47 ± 0.46d | 9.22 ± 0.25a | 35.05 ± 5.35d | 4.98 ± 0.28e | 22.03 ± 1.10a | 3.27 ± 0.07b | 32.14 ± 3.51bc | 1.67 ± 0.02f |
| C2 | 4.33 ± 0.56f | 9.13 ± 0.12ab | 41.59 ± 3.47c | 4.11 ± 0.54e | 5.61 ± 0.81f | 3.44 ± 0.09a | 33.88 ± 1.50b | 1.90 ± 0.06d |
| NG | 25.57 ± 1.19a | 8.12 ± 0.26d | 74.88 ± 8.01a | 36.31 ± 4.15a | 26.60 ± 3.12a | 2.71 ± 0.25d | 28.09 ± 1.23cd | 1.91 ± 0.06d |
| Groups | TN | PC | PR | PA | PS | ALP | UE | SC |
| SR | 0.07 ± 0.01e | 9.24 ± 1.51f | 2.67 ± 0.57cd | 8.20 ± 2.43e | 0.44 ± 0.23cd | 0.49 ± 0.07e | 44.24 ± 1.77e | 68.94 ± 11.50e |
| A1 | 0.08 ± 0.01d | 40.40 ± 5.73d | 4.90 ± 0.98b | 13.20 ± 4.03d | 0.95 ± 0.39b | 0.07 ± 0.01f | 39.53 ± 4.80f | 53.65 ± 7.17f |
| A2 | 0.09 ± 0.01c | 48.00 ± 7.07c | 5.33 ± 0.52a | 18.80 ± 4.50c | 1.20 ± 0.26a | 1.29 ± 0.15c | 46.32 ± 1.91d | 40.90 ± 4.70g |
| B1 | 0.09 ± 0.01c | 64.80 ± 4.90a | 3.00 ± 0.00c | 17.60 ± 4.83c | 0.54 ± 0.15c | 0.51 ± 0.05e | 52.65 ± 3.78c | 111.71 ± 12.03d |
| B2 | 0.07 ± 0.01e | 65.40 ± 4.73a | 3.00 ± 0.00c | 10.80 ± 3.81de | 0.81 ± 0.14bc | 0.55 ± 0.08e | 40.92 ± 2.18ef | 19.60 ± 3.82h |
| C1 | 0.07 ± 0.01e | 49.60 ± 6.17c | 2.00 ± 0.00e | 7.20 ± 1.56e | 0.52 ± 0.10cd | 0.40 ± 0.07e | 54.85 ± 2.86b | 165.58 ± 5.99c |
| C2 | 0.07 ± 0.01e | 59.20 ± 8.29b | 2.67 ± 0.58cd | 8.60 ± 1.91e | 0.32 ± 0.18d | 1.92 ± 0.31b | 59.38 ± 3.07a | 175.72 ± 23.00c |
| NG | 0.22 ± 0.01a | 32.00 ± 6.47e | 4.67 ± 1.12b | 89.33 ± 22.29a | 1.28 ± 0.28a | 11.62 ± 0.72a | 53.23 ± 1.79bc | 548.38 ± 58.93a |

Note, SM: soil moisture; AN: ammonium nitrogen; NN: nitrate nitrogen; AK: available potassium; AP: available phosphorus; TP: total phosphorus; TC: total carbon; TN: total nitrogen; PC: vegetation coverage; PR: species richness; PA: plant density; PS: Shannon-Wiener indices; ALP: alkaline phosphatase; UE: urease; SC: sucrase.


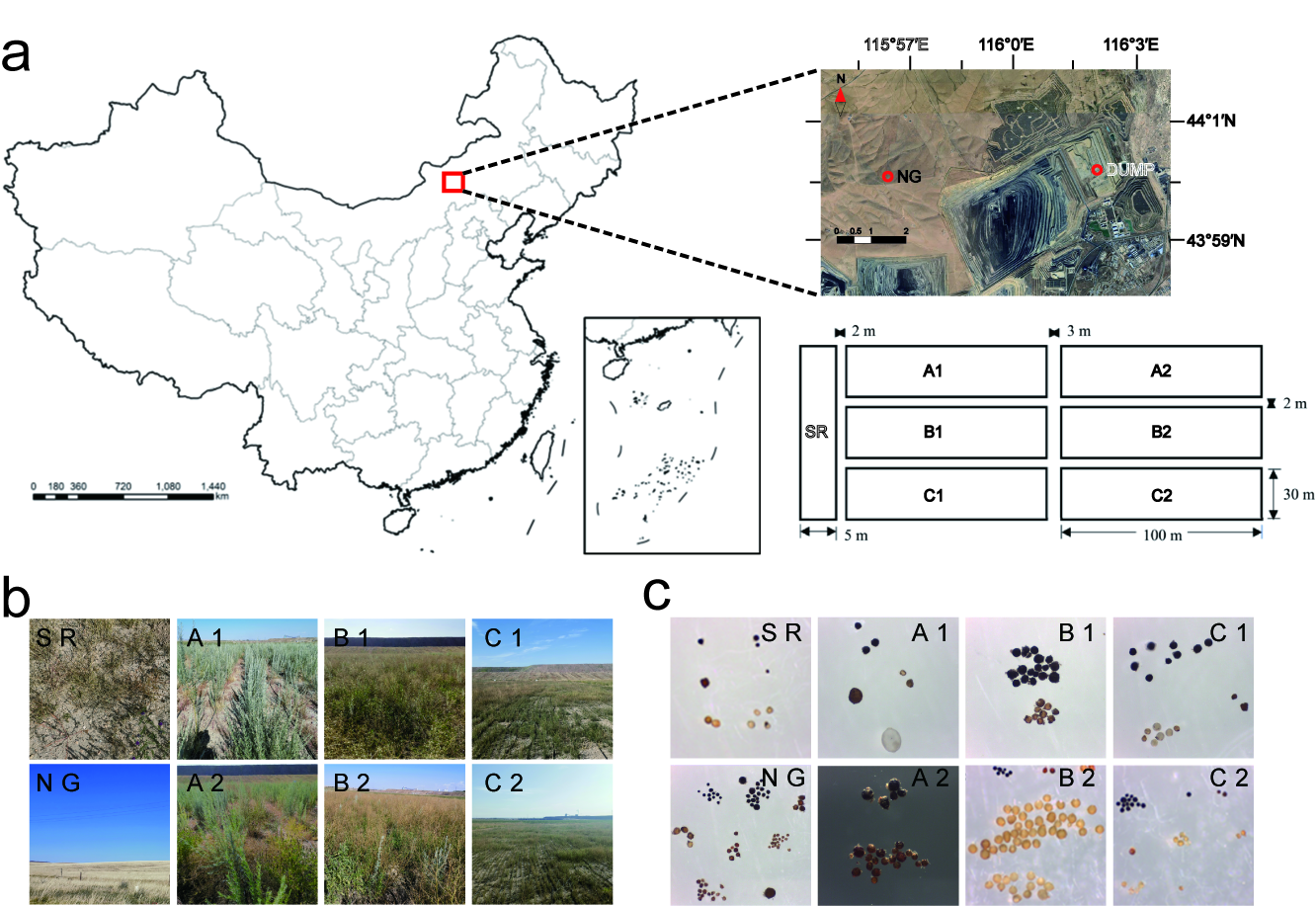


**Fig. S1.** Schematic diagram of the plot design. (a) design and spatial arrangement of the sampling plots; (b) field photographs showing the above-ground plant community composition in each plot; (c) soil AMF spore density observed under a 40× stereo microscope.


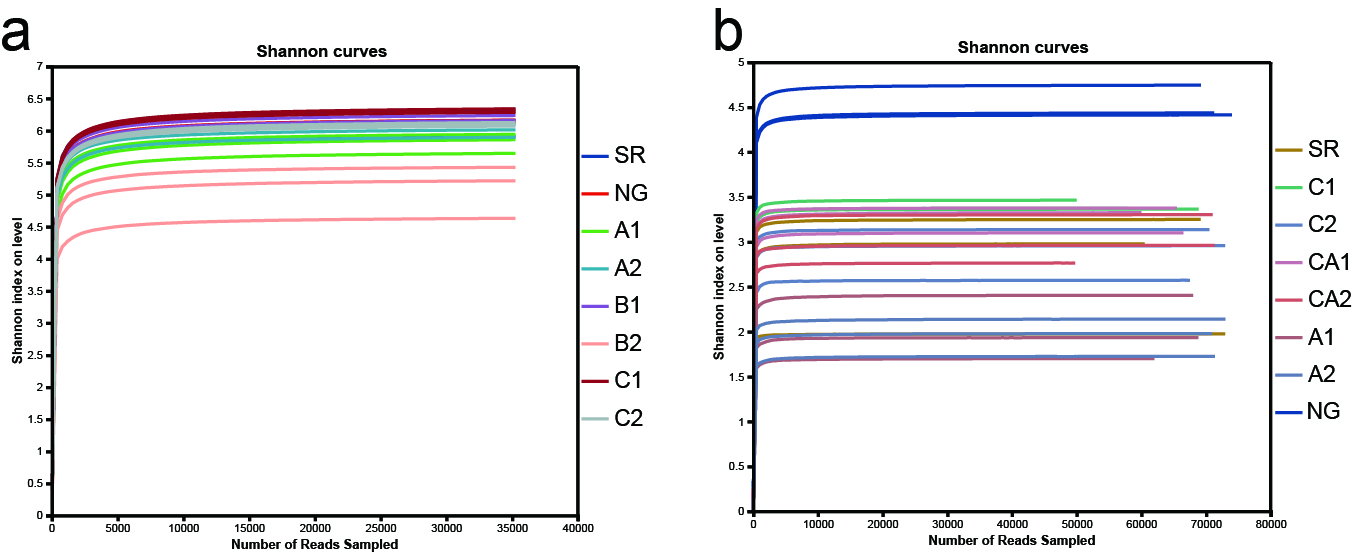


**Fig. S2.** The rarefaction curve analysis of the bacterial (a) and fungal (b) sequences. The Shannon index values of the OTUs and the number of reads were used to construct the curve.


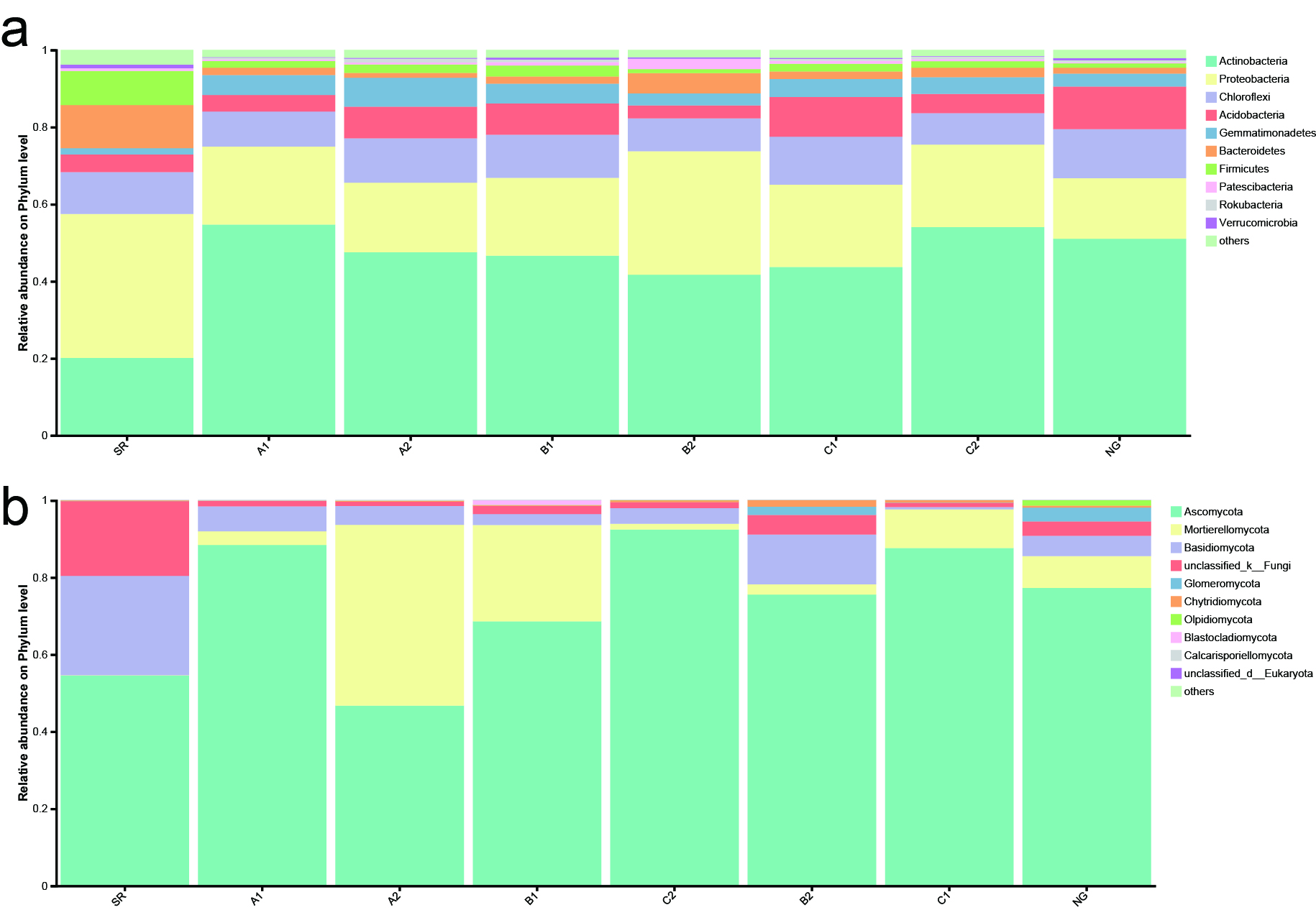


**Fig. S3.** Analysis of soil bacterial (a) and fungal (b) composition in studying areas.


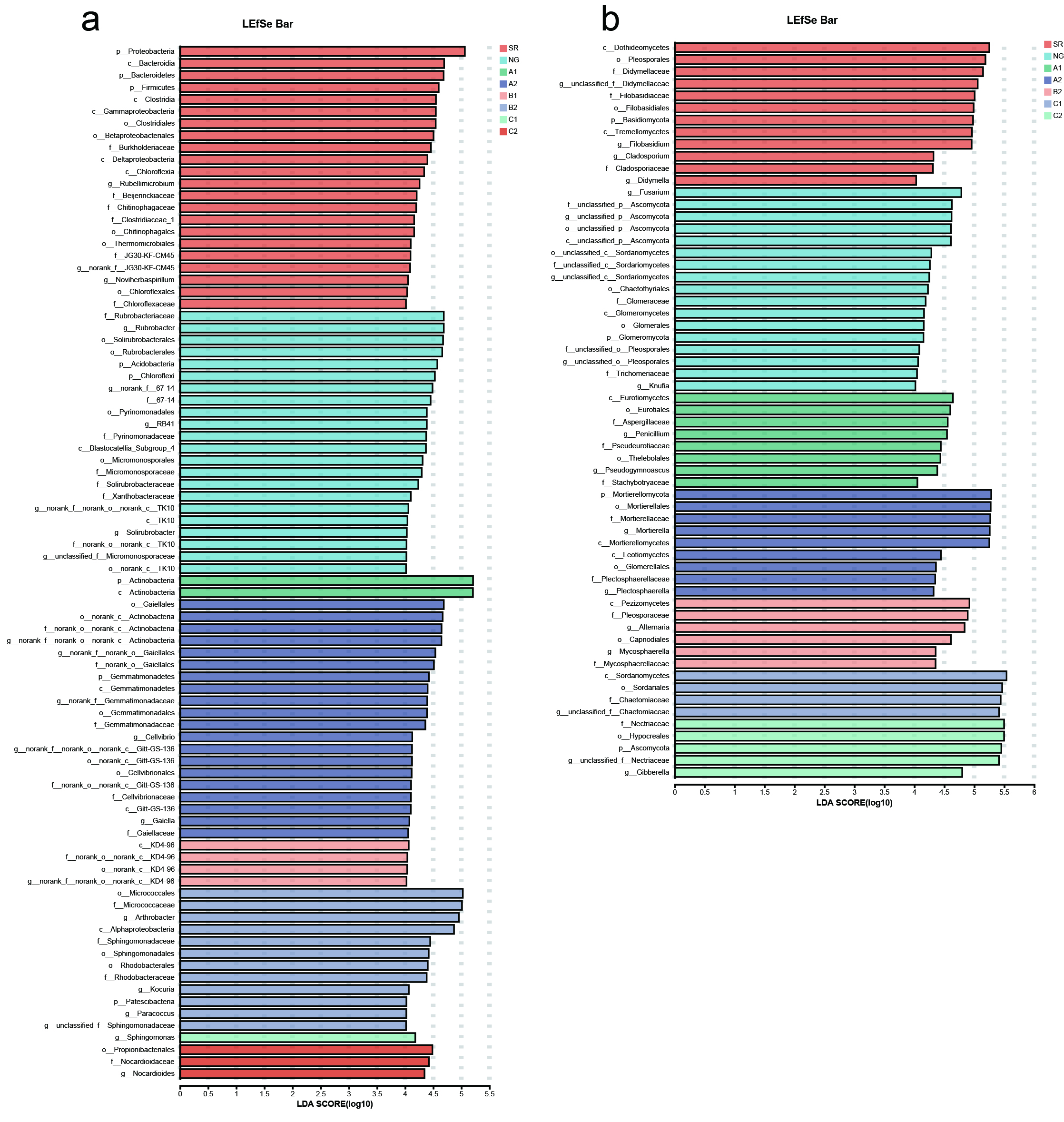


**Fig. S4.** Linear discriminant analysis effect size (LEfSe) of the bacterial (a) and fungal (b) communities with an LDA score higher than 4.0 and *P* values less than 0.05.
